# Evidence for the Use of Complementary and Alternative Medicine for Pelvic Inflammatory Disease: A Literature Review

**DOI:** 10.1155/2022/1364297

**Published:** 2022-01-19

**Authors:** Dongmei Wang, Yue Jiang, Jiaxing Feng, Jingshu Gao, Jinlan Yu, Jing Zhao, Pihong Liu, Yaguang Han

**Affiliations:** ^1^First Affiliated Hospital, Heilongjiang University of Chinese Medicine, Harbin, China; ^2^Heilongjiang University of Chinese Medicine, Harbin, China; ^3^Harbin Daoli District People's Hospital, Harbin, China; ^4^Heilongjiang Academy of Traditional Chinese Medicine, Harbin, China

## Abstract

Pelvic inflammatory disease (PID), a common infectious disease of the female reproductive tract, is mainly characterized by abdominal/pelvic pain and tenderness of the uterus, cervix, or adnexa on physical exam. In recent years, its incidence has gradually increased yearly due to numerous factors, including sexually transmitted diseases and intrauterine operations. Based on self-report of PID in the National Health and Nutrition Examination Survey (NHANES) 2013–2014 survey, PID impacts approximately 2.5 million women in the US during their reproductive age. Although empiric treatments such as antibiotics or surgery could alleviate the related symptoms of PID, its unsatisfactory obstetric outcome and high relapse bring heavy physical and psychological burden to women. Complementary and alternative medicine (CAM), a complementary therapy other than Western medicine with a complete theoretical and practical system, has been attached to importance in the world due to its remarkable efficacy. More people are accepting and trying to use CAM to treat gynecological diseases, including infertility, polycystic ovary syndrome, and PID, but its efficacy and mechanism are still controversial. This article reviews the previous literature systematically focusing on the effectiveness, safety, and mechanism of CAM in the treatment of PID to provide an evidence-based basis for the clinical application of CAM in patients with PID.

## 1. Introduction

Pelvic inflammatory disease (PID) refers to the inflammation of the female organs located at the upper genital tract and their surrounding tissues (uterus, fallopian tubes, ovaries, parauterine tissues, and peritoneum) caused by pathogen infection, often involving adjacent tissues. Inflammation can involve one site or spread to several sites simultaneously, mainly including endometritis, salpingitis, tubo-ovarian abscess, and pelvioperitonitis, with the highest incidence rate of salpingitis [1, 2]. The investigation in the recent ten years has shown that the prevalence of PID shows a significant upward trend. Approximately, 4%–12% of women during childbearing age worldwide suffer from PID [3]. In most developing countries, PID is more difficult to be controlled and effectively treated [4]. In China, the incidence rate of CPID in women who have more than 5 years of intercourse exceeds 20% [5], which seriously affects women's physical and mental health. From July 2013 to March 2014, a survey of 1010 women in a certain area of China showed that the prevalence rate of CPID was 5.8% and pointed out that abortion was one of the crucial factors of PID infection [6]. A survey of 1100 patients in a hospital found that the proportion of patients with reproductive tract infection was 4.36%, of which PID constituted 14.6% [7]. A census for diseases of married women in rural areas in China showed that the prevalence of PID accounted for 13.5% among all diseases on average, and in some areas, it could be highly 23.53% [8].

*Neisseria gonorrhoeae* and *Chlamydia trachomatis* are the main pathogenic microorganisms. In addition, some aerobic bacteria, anaerobic bacteria, viruses, and mycoplasma also participate in its occurrence. Pathogenic microorganisms are mostly mixed infections ascending from the vagina, leading to local tissue congestion, edema, inflammatory exudation, connective tissue hyperplasia, followed by irregular menstruation, abnormal leucorrhea, etc. [9, 10]. On most occasions, the symptoms of PID vary from none to severe. PID can be divided into two categories, namely acute PID and chronic PID. Chronic pelvic inflammatory disease (CPID), caused by PID not receiving prompt and effective treatment, can be accompanied by inflammatory lesions such as pelvic adhesions and tubal obstruction, leading to infertility, ectopic pregnancy, and chronic pelvic pain [11]. Due to the great variation of the clinical manifestations, the diagnosis of PID could not be completely accurate [12]. The diagnostic criteria recommended by the Centers for Disease Control and Prevention (CDC) are lower abdominal or pelvic pain and at least one of the following: adnexal tenderness or cervical motion tenderness or uterine tenderness [13].

PID has the characteristics of the long course, low cure rate, and high recurrence rate. Empirical therapies involves broad-spectrum combination regimens of antimicrobial agents to cover likely pathogens, and surgery can be performed if necessary [14]. Although the incidence and severity of PID in North America and Western Europe had reduced by using antibiotics in the past two decades, the ultimate efficacy was still not satisfactory. Complementary and alternative medicine (CAM), a supplement to conventional medicine, mainly includes the following methods: alternative medical systems, physical and mental intervention, biological therapy, manipulation and body-based methods, energy therapy, etc. [15]. The relevant report suggested that the rate of CAM application has reached 9.8%–76.0% globally [16]. At present, CAM has been widely utilized in female genital infections. Several randomized controlled trials (RCTs) have found that CAM has antibacterial and anti-inflammatory therapeutic effects, which could be effective in treating PID [17], but its efficacy and mechanism still exist in dispute. This article summarizes the efficacy of Chinese herbal medicine (CHM), acupuncture and moxibustion, pelvic exercises, hyperbaric oxygen therapy, and microwave physiotherapy in the treatment of PID and further explores their possible mechanism and safety in treating PID.

## 2. Researching the Overview of CHM in Treating PID

CHM, an integral part of traditional Chinese medicine (TCM), has a long history of treating diseases. In ancient times, Chinese people used certain animals, plants, and minerals to relieve the symptoms of diseases. Through long-term practice, the theories and methods of TCM have gradually formed and are recorded in written works, *such as* The Yellow Emperor's Inner Classic, Treatise on Cold Damage, and A Hundred Records on “Shen Nong's Classic of the Materia Medica”. Its basic characteristics are the concept of holism and syndrome differentiation and treatment. TCM has been widely used in different stages of disease, such as prevention, diagnosis, treatment, and rehabilitation. With the development of science and technology, TCM staff have provided a more theoretical basis for TCM to treat diseases by combining modern technology with original theories [18]. CHM has various dosage forms (e.g., pills, powders, granules, wines, tinctures, and ointments). It can also be processed into Chinese patent medicines. PID always threatens women, which needs to attract people's attention. For patients suffering from PID, adjuvant treatment of CHM seems to be in high demand. Studies have shown that CHM mainly administered by oral and retention enema has a definite curative effect and obvious advantages in treating gynecological diseases, especially for PID [19].

### 2.1. Clinical Effects of Oral Chinese Medicine Compound Formula (CMCF) in Treating PID

Clinically, oral CMCF combined with conventional therapy is used to treat PID. More and more RCTs showed that the combination therapy could greatly improve the clinical effective rate and reduce adverse reaction and recurrence rate [20]. Zhang and Zhang [21] found that CMCF combined with antibiotics in treating acute PID can shrink pelvic mass; reduce pelvic fluid, inflammatory factors, and adverse reactions; and improve immune function. Xiangli took the same method to treat PID. After treatment, the patients' symptoms such as fever, lower abdominal pain, and abnormal leucorrhea were significantly improved [22]. Feng's research showed that CMCF combined with antibiotics could effectively decrease the time of symptom relief and reduce the TCM syndrome score and the deepest diameter of pelvic effusion [23]. Wang and Gan added another CMCF (treatment arm) to treat PID based on CMCF combined with antibiotics (control arm) and found that the cure rate of treatment arm was higher than that of the other arm (*P* < 0.05). After treatment, TCM syndrome score, inflammatory mass diameter, pelvic effusion depth, serum ICAM-1, D-dimer, plasma viscosity, erythrocyte sedimentation rate, and platelet aggregation rate of treatment arm decreased more significantly than those of control arm. IL-2, IL-10, MMP-2, complement C3, and C4 were significantly higher than those of control arm [24]. Xiong et al. [25] also found that CHM combined with antibiotics has a more obvious effect on CPID. After treatment, the number of leukocytes, the proportion of neutrophils, and CD8+ and Th2 in treatment arm were significantly lower than those in the control group (*P* < 0.01); the levels of CD3+, CD4+, CD4+/CD8+, Th1, and Th1/Th2 were significantly higher than those in the control group, and the total effective rate was significantly higher than that in the control group (*P* < 0.05). Xiong [26] found that the serum estradiol (E2), cancer antigen 125 (CA125), and hemorheological indicators of PID patients treated with CMCF combined with antibiotics were lower than those treated with antibiotics alone. Besides, some studies have reached the same conclusion [27–30]. In a double-blind, multicenter, placebo-controlled clinical trial, 155 patients with PID were randomly assigned into treatment arm (*n* = 77) and control arm (*n* = 78). The treatment arm was given CMCF combined with antibiotics placebo in the first two weeks. Subsequently, the patient received oral CMCF merely for the remaining weeks. While the control arm was administered two kinds of medicines together with CMCF placebo in the first two weeks and CMCF placebo for the remaining weeks. The follow-up results after treatment showed that the cure rate together with the effective rate of TCM syndrome in treatment arm was 81.82%, significantly higher than 67.95% in control arm (*P* < 0.05), which demonstrated that the oral CMCF could reduce the antibiotic dosage required for PID treatment and improved symptoms in PID patients [31]. In addition, some studies are comparing the efficacy of oral CMCF with conventional therapy. Zhou and Chen [32] compared the efficacy of CMCF with antibiotics in treating endometritis by observing the morphological changes of the endometrium through hysteroscopy and performing an endometrial biopsy at a fixed position. The results showed that the markedly effective rate of clinical symptoms in the CMCF group was 91.3%, significantly higher than that of the control group 60.0% (*P* < 0.05), and plasma cell CD38 infiltration of the endometrial stroma was significantly decreased, the positive expression of MUC-1 increased, and the expression of HIF-1*α* was reduced, indicating that CMCF has a better therapeutic effect on chronic endometritis compared with antibiotics. The clinical effects were also not completely consistent among different oral CHMs. Wu et al. compared the clinical efficacy of two CMCFs (Tongluo Qingre Decoction and Gexia Zhuyu Decoction) on PID through an RCT and found that the effective rate of Tongluo Qingre Decoction was higher than that of Gexia Zhuyu Decoction (*P* < 0.05). After treatment, compared with the patients administered with Gexia Zhuyu Decoction, those taken Tongluo Qingre Decoction had lower TCM syndrome scores, and the patients' peak systolic flow velocity of uterine blood flow levels was higher (*P* < 0.05), and the flow resistance index and pulsatility index were lower (*P* < 0.05) than those administered with Gexia Zhuyu Decoction. The study concluded that Tongluo Qingre Decoction had better efficacy in treating PID and was more conducive to improving uterine blood microcirculation [33]. Huang randomized 180 CPID patients to CMCF arm, physiotherapy arm, and combination therapy arm. Patients in CMCF arm were given oral CMCF, physical therapy arm was given physical therapy, and combined treatment arm was given oral CMCF combined with physical therapy. After treatment, the effective rate of combined therapy arm was 98.3%, which was significantly higher than that of CMCF arm (86.7%) and physical therapy arm (76.7%), thus demonstrating that oral CMCF combined with physiotherapy could significantly improve the effect of CPID patients, strengthen the immunity of patients, and reduce inflammation. In addition, it can improve blood rheology indicators as well [34]. Furthermore, studies have found that the combination of CMCF and antibiotics for PID is not as good as expected. Lan et al. combined CMCF with antibiotics to treat CPID. The total effective rate of treatment arm was 95.00%. Although the levels of C-reactive protein and IL-10 were significantly lower than those in control arm after treatment, the incidence of adverse reactions and recurrence rate at 3 and 6 months were similar, without any significant difference [35].  Table 1 lists some of the above study protocols and some unmentioned protocols [24–38].

### 2.2. The Mechanism of Oral CMCF in Treating PID

Extensive research has proved that the mechanism of CHM in treating PID is associated with inflammatory cells, inflammatory factors, and related pathways. Zhang et al. found that the administration of CHM to CPID-like rats could decrease the levels of IL-2, IL-6, IL-10, TNF-*α*, and TGF-*β*1 in serum, increased the mRNA of P53, Fas/FasL, and MMP-2 mRNA in the uterus, while decreased TGF-*β* 1 mRNA, and inhibited NF-*κ*B p65 in the uterus and ovary tissue. These results indicated that the possible mechanism of CHM in treating CPID was achieved by inhibiting the inflammatory response, inducing the inflammatory cell apoptosis, and downregulating serum inflammatory cytokines [36]. A study conducted by Zou et al. [37] has suggested that CHM could inhibit the infiltration of lymphocytes and neutrophils in the fallopian tubes of PID-like rats, reduce the release of IL-1*β*, IL-6, IL-8, and MCP-1, promote the production of LXA4, and found that CHM could regulate LPS-stimulated NF-*κ*B signaling activity and promote FPR2 expression in THP-1 cell line, therefore contributing its anti-inflammatory effect. The experimental results of Zheng et al. showed that compared with PID-like rats not administered with CHM, the levels of serum IL-6, IL-8, and TNF-*α* in PID-like rats treated with CHM significantly decreased. It was concluded that CHM had strong anti-inflammatory and anti-infective effects, and could be used to treat PID and relieve pain [38]. Sun [39] explored the efficacy of CHM combined with levofloxacin in treating CPID. 120 patients were randomized into control arm and observation arm. The control arm was given levofloxacin lactate injection, and the observation arm was combined with CHM on the basis of control arm. After treatment, the levels of IL-2 and IL-10 in the observation arm were higher than those in the control arm, and the levels of CRP and TNF-*α* were lower than those in the control arm (*P* < 0.05). Xia et al. [40] analyzed endogenous small-molecule metabolites in the serum of rats after CHM treatment based on gas chromatography-mass spectrometry (GC-MS)-based metabolic profiling method combined with multivariate statistical analysis, such as PCA, PLS-DA, and OPLS-DA. The results showed that CHM treatment could significantly improve the inflammatory pathological characteristics and tissue damages of model rats. Based on the principle of VIP > 1 and *P* < 0.05, six different metabolic biomarkers, i.e., L-valine, L-isoleucine, L-threonine, butanedioic acid, serine, and D-glucose, were identified, and their contents were significantly reversed after administration. Further analysis of the metabolic pathways in the KEGG database showed that CHM could achieve this effect through the metabolism of glycine, serine, and threonine, biosynthesis of aminoacyl tRNA, and biosynthesis of valine, leucine, and isoleucine. Li et al. [41] used the network pharmacology method to screen targets and found that the PTGS2 target in the arachidonic acid (AA) pathway was significantly correlated with CPID, which further confirmed that CHM could reduce the development of CPID by regulating PTGS2 target.

### 2.3. Clinical Effects and Mechanism of CHM Retention Enema (CHMRE) in Treating PID

CHMRE evolved from the enema method in “Treatise on Cold Damage” is one of the most commonly used external treatment methods and also known as anorectal administration. Pouring CHM into the rectum and keeping it for four to five hours to be absorbed fully by intestinal mucosa to treat the disease. In line with physiological and anatomical characteristics of the female pelvic cavity, the transrectal administration of CHM can make the drug absorbed through the mucosal venous plexus [42–44], directly arrive at the lesion, accurately and quickly exert the drug effect, improve local microcirculation, and promote the absorption of effusion and mass [45, 46]. The possible mechanism of CHMRE in treating PID is related to its ability to improve blood rheology, reduce oxidative stress response, reduce inflammation, etc. [47]. Due to its simplicity and convenience, CHMRE has a wide range of clinical applications. In addition, compared with conventional therapies, the medicine enters the internal iliac vein through the inferior rectal vein and/or anal vein, then finally into the systemic circulation, which can reduce the irritation of the digestive system, avoid the first pass elimination to improve the bioavailability of drugs, and reduce the damage to the liver and other organs, therefore greatly reducing the adverse effects and side effects [48–50]. To assess the efficacy of CHMRE in treating PID, Liang and Ling carried out a comparative study on 184 patients who suffered from sequelae of PID and found that the total effective rate of CHM staining therapy combined with CHMRM arm was higher than that of abdominal ultrasound drug delivery therapy arm, with statistically significant difference (*P* < 0.05) [51]. A clinical study [52] on a larger number of people has reported that compared with 55 patients with routine anti-inflammatory treatment, patients with CHMRE combined with anti-inflammatory treatment had a significantly higher effective rate, and serum TNF, IL-2, and IL-10 levels were statistically significant. Research conducted by Shao demonstrated that the *CHMRE* has a significant effect on PID, especially in terms of pain relief, recurrence reduction, and prevention of long-term complications [53]. More recently, numerous clinical studies have drawn the same conclusion [54–56]. Some researchers utilized *CHMRE* after hysteroscopy, which effectively reduced the levels of TNF-*α*, IL-6, and IL-8, improved the unobstructed fallopian tube, and relieved abdominal pain and other symptoms, thereby promoting the recovery of fertility [57–60]. *CHMRE* could also reduce the recurrence rate of PID for half a year or more [61, 62]. Based on the above-mentioned research works, CHMRE has significant efficacy in treating PID with a low incidence of adverse reactions, which is worthy of clinical application. However, it should be noted that the temperature of CHM should be between 39 and 41°C to prevent intestinal spasms or scald of intestinal mucosa caused by inappropriate temperature. Of course, CHMRE also has its shortcomings. For very few patients, the intolerance of it manifested as severe diarrhea and abdominal distension affects their quality of life seriously. In addition, for patients after intestinal tumor surgery, since the normal barrier function of the intestine has been broken, special attention should be paid to the medication. The above studies are listed in Table 2.

### 2.4. The Application of Chinese Medicine Monomer in Treating PID

Apart from CMCF, Chinese medicine monomers are also generally used in treating PID. Sargent gloryvine stem (SC) and *Patrinia scabiosifolia* (PS) have the effects of clearing heat and resolving toxins, invigorating blood, and dissolving stasis, which are the key to treating PID. Modern pharmacological studies have suggested that SC and PS contained various active ingredients such as phenols, flavonoids, phenylpropenes, and triterpenes, which have antioxidant, antibacterial, anti-inflammatory, and antiviral effects [63]. Their active ingredients act on key targets such as VEGFA, VWF, IL6, TNF, and NFKB1, therefore regulating AGE-RAGE, FA, Toll-like receptors, PI3K/Akt, NF-*κ*B, apoptosis, and cancer signaling pathways. Zhang Y used Chinese medicine *Smilax china* L. to treat PID. Its active ingredient *Smilax china* polysaccharide is considered to have an anti-inflammatory effect. Through extraction, purification, and structural identification, it was discovered for the first time that *Smilax china* L. polysaccharide can be purified to produce polysaccharides SCLP1 (*Smilax china* L. polysaccharide 1,42.1 kDa) and SCLP3-2 (*Smilax china* L. polysaccharide 3,2, 16.8 kDa), which structure had been identified by chemical and spectral analyses. The results showed that SCLP1 and SCLP3-2 could inhibit the production of NO and IL-6 in RAW264.7 cells stimulated by LPS through NF-*κ*B and MAPKs (ERK1/2, JNK) pathways [64]. Kong D studied the therapeutic efficacy and potential mechanism of Asian acid (AA) on PID-like rats. The results showed that AA treatment significantly reduced the overproduction of cytokines and chemokines and inhibited MPO activity, NLRP3 inflammasome, activation of NF-*κ*B and caspase-3, and oxidative stress, indicating that AA had stronger anti-inflammatory and antioxidant effects on PID-like rats. Its anti-inflammatory mechanism may be related to the inhibition of NLRP3 inflammasome activity and the NF-*κ*B pathway [65].

## 3. Researching the Overview of Acupuncture and Moxibustion in Treating PID

Acupuncture and moxibustion, a widely practiced traditional medical system that existed for more than 3,000 years, is considered to be rooted in naturalistic theories compatible with Confucianism and Taoism [66–68]. Since the reform and opening up, acupuncture and moxibustion have been gradually accepted by Western countries, which have been playing an important role in the internationalization of TCM. With its unique advantages (e.g., simple operation, durable, and strong quantity of stimulus), acupuncture and moxibustion have been recognized and applied in 183 countries around the world. There are about 200,000 practitioners [69–73]. In clinic, acupuncture and moxibustion are usually divided separately. Guided by the theory of meridians and acupoints, practitioners use needles and artemisia as tools and materials to stimulate specific parts of the body through needles or burning artemisia to adjust the balance of Yin-Yang, therefore achieving the purpose of treating and preventing diseases. PID usually has longer treatment courses and a relatively low cure rate due to its complicated etiology. As a branch of CAM, acupuncture can enhance immunity and relieve the lower abdominal pain of PID patients. Moreover, it is simple in operation, available, and accepted by patients because of its fewer side effects. Due to such superiorities, acupuncture and moxibustion have aroused the attention of many gynecologists, so a series of clinical and animal studies have been carried out to evaluate the effect and therapeutic mechanism in PID.

### 3.1. Clinical Effect of Acupuncture in Treating PID

Acupuncture can effectively treat more than 500 types of diseases. In 1971, the acupuncture anesthesia test was successful, which set off an upsurge of acupuncture anesthesia. Later, when US President Nixon visited China in 1972, the accompanying journalists experienced acupuncture for analgesia, which made medical workers worldwide arouse great interest in acupuncture for treating diseases [74, 75]. Acupuncture has a long history of treating gynecological diseases, and abundant studies have shown that acupuncture is beneficial, particularly in treating CPID [76, 77]. There are many categories of acupuncture treating CPID, including filiform acupuncture, warming-acupuncture, etc. Selecting appropriate acupuncture based on patients' clinical manifestations is conducive to their recovery [78]. Before the 1980s, there were fewer reports on the related studies about acupuncture in PID, let alone the large-sample clinical RCTs. In 1989, Wang [79] randomly assigned 95 patients with CPID into treatment arm (electroacupuncture (EA) combined with moxibustion) and control arm (antibiotic). The results showed that the therapeutic effect of EA combined with moxibustion was better than antibiotics (*P* < 0.05). This was the first clinical trial to show that acupuncture was effective in treating PID. Subsequently, in 2008, Zhen and Wang [80] randomized 85 CPID patients into the warming-acupuncture arm and the CHM arm and concluded that the effective rate was 95.6% versus 77.5%, respectively, with a significant statistical difference (*P* < 0.05). During the follow-up, the recurrence rate of the treatment arm was 8.7%, while the other arm was 22.2%. Zhang [81] compared the efficacy of Western medicine with acupuncture combined with CHMRE in treating patients with acute PID. The results showed that the effective rate of acupuncture combined with CHMRE was higher than that of Western medicine (*P* < 0.05). Therefore, it is concluded that acupuncture combined with CHMRE is effective in treating acute PID, and the two therapies have a synergistic effect, which can better relieve pain and symptoms. Wu et al. [82] investigated the effect of acupuncture on the inflammation and symptoms of patients suffering from acute PID. The efficacy of observation arm was better than that of control arm (95.24% vs 81.08%, *P* < 0.05). After treatment, the levels of TNF-*α* and CRP in the serum were significantly decreased (*P* < 0.05). Shi [83] also got a similar conclusion. Liu et al. [84] conducted a multicenter RCT. The results showed that acupuncture combined with ibuprofen sustained-release capsule can effectively improve the symptoms and signs of patients with chronic pelvic pain caused by PID and improve their quality of life, which was more effective than ibuprofen sustained-release capsule alone. Several systematic reviews and meta-analyses of acupuncture treatment of PID have been published successively. Although these studies had varying degrees of bias risk, the conclusions still provided strong evidence for acupuncture as supplements to substitute Western medicine to treat PID. Zheng et al. [85] used data mining technology to analyze the rule of acupoint selection in treating CPID. The results showed that acupoints of 14 meridians were mainly selected for treating CPID, of which CV4 (168 times) and SP6 (155 times) were used most frequently, with the proportion as high as 76.02% and 70.14%, respectively. According to the analysis of the regularity of the use of extra meridian acupoints, EX-CA1 has the highest frequency followed by EX-B7 and Changyi. He et al. [86] conducted a meta-analysis on the efficacy and safety of acupuncture in treating CPID. The results showed that, in terms of efficacy, the total effective rates of acupuncture and its combination with medicine were higher than that of medicine alone; there was no significant difference in the incidence of adverse reactions in acupuncture arm compared with the control arm. We have listed the 10 most commonly used acupoints for the treatment of PID in Table 3.

### 3.2. The Mechanism of Acupuncture in Treating PID

Studies have shown that acupuncture induces reactions such as activation of nerve, endocrine, and immune signaling pathways by stimulating skin tissue [87]. There are many pathogenic factors of PID [88], including a variety of microbial infections, decreased autoimmunity, and pelvic floor muscle dysfunction. The main mechanisms of acupuncture in treating PID are as follows: first of all, acupuncture can promote blood circulation, increase the permeability of the cell membrane, and accelerate the absorption of inflammation, thereby treating PID; second, acupuncture can enhance immunity by stimulating health-care acupoints and specific acupoints, thereby preventing the occurrence of PID and promoting the recovery of the disease; and finally, acupuncture treats PID by improving persistent pain in the lower abdomen. Now, we will discuss these three aspects in detail in the following sections.

#### 3.2.1. Acupuncture Could Promote Blood Circulation and Accelerate Inflammation Absorbed

Acupuncture can promote blood circulation, dilate blood vessels and lymphatic vessels, increase the permeability of cell membranes, exhibit an anti-inflammatory effect to a certain extent, and promote the dissipation and absorption of pathological products and inflammatory exudates [89–91]. Shi [83] randomly divided 70 CPID patients into control arm (conventional therapy) and observation arm (conventional therapy combined with acupuncture). After treatment, the imageology showed that the time of inflammation absorbed and abdominal pain relief in the observation arm were shorter than those of control arm, with a statistically significant difference (*P* < 0.05). The levels of TNF-*α*, hs-CRP, and IL-6 in the two arms were decreased, and the observation arm was lower than the control arm (*P* < 0.05). Therefore, acupuncture can effectively increase the absorption of inflammation, which could be recommended for the treatment of PID [71].

#### 3.2.2. Acupuncture Can Improve Immunity

The body mainly relies on the immune system to fight infection, with innate immunity in the early stage and acquired immunity in the later stage. Under the stimulation of pathogenic bacteria, the body activates its autoimmune system and releases plenty of cytokines and chemokines through a series of signal transduction, thereby killing the pathogenic bacteria. In the reproductive system, the innate immune system, namely Toll-like receptors (TLRs), is the first to be activated [91]. It plays an important role in the host's antimicrobial infection by regulating innate and acquired immunity and is the key to linking infection, inflammation, and injury [92]. Studies have shown that the variation of Toll-like receptors is closely related to the occurrence and development of PID [93]. Huang conducted an RCT on 80 patients with PID, and the conclusion showed that the application of acupuncture combined with acupoint injection in treating patients with CPID can improve patients' immunity, enhance efficacy, and reduce relapse [94]. Research by Jiang Yu et al. pointed out that acupuncture at Dong Qi points (a special acupuncture therapy popular in Taiwan, Europe, and the United States) can enhance the immunity of patients with CPID [95].

#### 3.2.3. Acupuncture Can Relieve Lower Abdominal Pain

Pain located at the lower abdomen and lumbosacrum is the most common symptom of PID, which affects the life quality of most patients [96]. Numerous studies have confirmed that acupuncture can effectively relieve pain [88]. The experiment by Wang explored the efficacy of warming-needle moxibustion in treating CPID with the type of qi stagnation and blood stasis and concluded that warming-needle moxibustion could significantly eliminate pain and other clinical manifestations of CPID patients and relieve their anxiety and depression [97]. Gr [98] explored the efficacy of acupuncture combined with moxibustion in treating CPID. The results showed that, after treatment, the NRS pain score of the treatment arm was lower than that of the control arm (*P* < 0.05), and the total effective rate was higher than that of the control arm (*P* < 0.05).

### 3.3. The Safety of Acupuncture in Treating PID

Acupuncture is relatively safe on most occasions, only improper operation performed by unqualified acupuncturists can cause adverse reactions such as faint during treatment, stuck needle, bending of the needle, needle breakage, and hematoma. Compared with conventional therapies, treating PID with acupuncture has fewer adverse reactions, most of which are skin erythema, bruising, and pain [99]. These problems usually can be reduced or avoided by careful operation. According to an RCT, the incidence of adverse reactions in the acupuncture arm was 0.75%, which was significantly lower than 25.00% in the control arm (*P* < 0.05) [84]. In another clinical trial of 62 patients with pain treated by acupuncture, only 5 patients had adverse events [100]. Compared with significant efficacy, most minor adverse reactions can be ignored [101].

At present, a large number of clinical trials have shown that acupuncture has a clear effect on PID, and the mechanism is also relatively clear. However, there are still some limitations such as insufficient sample size and lack of high-quality evidence. Therefore, large-scale RCTs are needed to further verify the efficacy and mechanism of acupuncture in treating PID.

### 3.4. The Efficacy and Mechanism of Moxibustion in Treating PID

As a natural therapy, moxibustion is to fumigate acupoints by igniting moxa-sticks made from artemisia to treat diseases. Its mechanism is similar to acupuncture, and they have a synergistic effect in treating diseases. Moxibustion is commonly used to treat gynecological diseases such as PID, dysmenorrhea, premature ovarian failure, blocked fallopian tubes, and infertility [102–106]. Among different types of moxibustion, heat-sensitive moxibustion and medicine-separated moxibustion are most frequently utilized to treat PID. Heat-sensitive moxibustion, also known as “heat-sensitive suspension moxibustion,” refers to using the heat generated by burning moxa-sticks to stimulate heat-sensitive moxibustion sensation (e.g., heat permeability, heat expansion, heat transfer, local heatless but remote heat, nonheat sensation) to promote pelvic blood circulation, accelerate the absorption, and dissipation of inflammation, thereby treating PID [107, 108]. It has the characteristics of without touching the body, injury, side effects, etc. Yin's RCT compared the efficacy of acupuncture and heat-sensitive moxibustion in treating CPID. The results showed that the efficacy of heat-sensitive moxibustion was better than that of acupuncture, and the effective rates were 93.33% vs 77.78% (*P* < 0.05) [109]. Wang et al. randomly assigned 208 cases with TCM syndrome type of qi stagnation and blood stasis CPID into Xuefu Zhuyu capsule arm and heat-sensitive moxibustion Ren Du meridian combined with Xuefu Zhuyu capsule arm. The results showed that the effect of heat-sensitive moxibustion on Ren Du meridian combined with Xuefu Zhuyu capsule was better. Meanwhile, it can significantly reduce the levels of CA125 and IL-8 and increase the level of TGF-*β*1 in serum. Therefore, heat-sensitive moxibustion plays an important role in treating CPID [110]. Medicine-separated moxibustion is a combination of moxibustion, acupoints, and medicine. Medicine-separated moxibustion is an effective treatment for CPID with TCM syndrome type of cold-damp stagnation, which fully combines the efficacy of TCM with the heat produced by moxibustion. Because the skin around the umbilical cord is thin, it is easier for the drug to reach the lesion directly, thereby improving the arterial blood supply of the uterus, promoting the absorption of pelvic inflammation, and achieving the purpose of treating disease [111]. Some studies have shown that moxibustion can promote metabolism, increase hematopoiesis of erythrocytes, leukocytes, hemoglobin, and enhance immunity [112, 113]. In addition, studies have confirmed that moxibustion could significantly reduce the levels of CRP, IL-2, IL-6, and TNF-*α* in the serum of CPID patients and could effectively control the patient's infection status [114, 115]. At present, there are few adverse reactions related to moxibustion in treating PID. The adverse reactions could be that the burning moxa-sticks fall off then scald the skin and the symptoms such as fever, thirst, and skin pruritus after moxibustion. In short, moxibustion has its unique advantages in treating PID. However, there still lacks research on moxibustion's heat-sensitive effect and thermal radiation effect, which should be the focus of future research.

## 4. The Overview of Other CAMs in Treating PID

In addition to acupuncture and CHM, pelvic exercises, hyperbaric oxygen therapy, and other therapies can also treat PID.

### 4.1. The Application of Pelvic Exercises in Treating PID

Pelvic exercise belongs to aerobic exercise, which can increase the tension of pelvic ligaments and blood vessels, improve pelvic blood circulation, and promote inflammatory absorption [116]. In addition, pelvic exercise can enhance the pelvic floor ligament and muscle strength, promote local oxygen uptake, and relieve symptoms such as lumbosacral pain and lower abdominal distension [117, 118]. Pelvic exercise, the behavioral therapy in psychotherapy, can improve symptoms, at the same time, it can also help patients adjust their mentality in time, establish the confidence to cure the disease, ensure patient compliance with treatment, and improve clinical efficacy [119]. A large number of studies have found that pelvic exercise can effectively reduce the level of immune factors, such as TNF-*α*, CRP, IL-2, IL-6, IL-10, and MDA, and help to relieve abdominal pain. It is safe and worthy of clinical application [120–123].

### 4.2. The Application of Hyperbaric Oxygen Therapy in Treating PID

Hyperbaric oxygen therapy has the function of increasing blood oxygen content and oxygen partial pressure and improving the state of systemic organs, which can be used to treat gynecological diseases such as infertility, premature ovarian failure, and PID [124–126]. Studies have shown that hyperbaric oxygen has a bactericidal effect, especially on anaerobic bacteria, and can inhibit the growth of aerobic bacteria [127, 128]. Other studies have shown that hyperbaric oxygen plays a role in the treatment of PID by downregulating CRP and inflammatory cytokines [129]. Hyperbaric oxygen can also improve the body's immunity and reduce the recurrence and complications of PID [130]. Many pieces of literature reported that hyperbaric oxygen combined with the drug could increase its effect, which was worthy of further clinical application [131, 132].

### 4.3. The Application of Microwave Physiotherapy in Treating PID

Microwave physiotherapy, complementary therapy for PID, can dilate blood vessels, accelerate blood circulation and metabolism, and improve tissue nutrition. A large number of experiments have confirmed that microwave physiotherapy combined with TCMRE can accelerate the absorption of pathological and inflammatory products, promote local blood circulation, and significantly improve inflammatory factors and hemorheological indicators [133–136]. Microwave physiotherapy can also improve the patients' ability to prevent disease and effectively prevent the recurrence of PID [137, 138]. Some RCTs have shown that mild moxibustion combined with microwave physiotherapy can promote blood circulation, accelerate metabolism, and effectively alleviate the symptoms of CPID patients with qi stagnation and blood stasis [139–141].

### 4.4. The Application of Cupping in Treating PID

Cupping is an external therapy of TCM to prevent and treat diseases. A negative pressure environment is formed in the cupping through methods such as combustion and suction. After cupping is adsorbed on acupoints or corresponding parts of the body, the local tissue would be congested, thereby achieving the purpose of warming the channels and unblocking the collaterals, relieving swelling and pain, drawing out the toxin, and expelling pus [142]. Modern studies have shown that cupping can regulate the function of the nervous system, improve the function of phagocytes, and promote blood circulation. Therefore, it has been proven to be one of the unique ways to treat PID [143]. The negative pressure stimulation produced by cupping can not only promote pelvic blood circulation, increase metabolism, be beneficial to the absorption of inflammation, and repair damaged tissues but also enhance immunity, shorten the course of treatment, and reduce the recurrence rate [144–146].

### 4.5. The Application of Ozone Therapy in Treating PID

Ozone is a highly active molecule with antioxidant activity, which can be used as a complementary alternative therapy for PID [147–149]. Through animal comparative studies, Wei et al. found that O3 can treat PID by inhibiting the necrosis of endometrial epithelial cells and reducing the inflammatory response, which provides a new target for the treatment of PID [150]. Escandón et al. [151] researched that ozone can reduce endometritis and improve the fertility of dairy cows. Ozone therapy is becoming a new adjuvant therapy for female reproductive health.

## 5. Summary

In recent years, the incidence of PID has reached as high as 12%, and the risk of depression secondary to PID has also increased yearly [152]. Clinical treatment is mainly focusing on antibiotics. However, antibiotic abuse is severe, and the potential risks such as flora imbalance, bacterial resistance, super bacteria production, and increased adverse reactions have become increasingly prominent. The most commonly used CAMs for treating PID are acupuncture and CHM. In addition, this review also mentioned the application of pelvic exercises, hyperbaric oxygen therapy, microwave physiotherapy, cupping, and ozone therapy in PID. In summary, the merits of CAM in the treatment of PID mainly include the following: (1) CAM effectively alleviates the symptoms caused by PID and accelerates the disappearance time of symptoms; (2) CAM can greatly improve clinical effective rate and reduce adverse reactions and recurrence rates; and (3) the efficacy produced by CAM can replace antibiotics and reduce the dose of antibiotics required for PID treatment. As an adjuvant treatment of PID, CAM's controversial efficacy and mechanism have aroused the horizon of many Western medical scholars. At present, due to the limitations such as small sample size, low quality, and lack of uniform standards, the effectiveness of CAM in the treatment of PID has been controversial. Therefore, we are looking forward to more high-quality research on CAM in the treatment of PID to provide a more convincing basis for CAM treatment of PID.

## Figures and Tables

**Table 1:** The RCTs of effective CHM formula for PID with oral administration.

Study ID	Design	Sample size	Interventions	Outcomes	Composition	Limitations
[21]	RCT	144	Treatment arm: Dachaihu Decoction + cefoxitin + doxycycline hyclate Control arm: cefoxitin + doxycycline hyclate	Treatment arm: TFR, 97.22% (70 of 72) Control arm: TFR, 84.72%^*∗*^ (61 of 72)	Dachaihu Decoction: White peony root (Bai shao), Corydalis tuber (Yan hu suo), Herba Patriniae (Bai jiang cao), *Bupleurum* (Chai hu), Coix seed (Yi yi ren), rhubarb root and rhizome (Da huang), dandelion (Pu gong ying), dried ginger rhizome (Gan jiang), fruit of citron or trifoliate orange (Zhi shi), *Pinellia ternata* (Ban xia), Trogopterus dung (Wu ling zhi), Radix scutellariae (Huang qin), Jujube (Da zao) , Delavay honeysuckle (Jin yin hua), Monkshood (Fu zi)	Not mentioned blindness and drop-out rate

[22]	RCT	360	Treatment arm: Gongying Tuling Decoction + moxifloxacin Control arm: moxifloxacin	Treatment arm: TFR, 91.67% (165 of 180) Control arm: TFR, 84.44%^*∗*^ (152 of 180)	Gongying Tuling Decoction: dandelion (Pu gong ying), bearded scutellaria (Ban zhi lian), Coix seed (Yi yi ren), glabrous greenbrier rhizome (Tu fu ling), white Atractylodes rhizome (Bai zhu), Pinellia rhizome (Ban xia), Amur cork-tree bark (Huang bai), Corydalis tuber (Yan hu suo), Atractylodes rhizome (Cang zhu)	Not mentioned blindness and drop-out rate

[23]	RCT	78	Treatment arm: Hongteng Baijiang Decoction + levofloxacin + metronidazole Control arm: levofloxacin + metronidazole	Treatment arm: TFR, 94.87% (37 of 39) Control arm: TFR, 79.49%^*∗*^ (31 of 39)	Hongteng Baijiang Decoction: Herba Patriniae (Bai jiang cao), weeping forsythia capsule (Lian qiao), Delavay honeysuckle (Jin yin hua), Sargentodoxa cuneata (Hong teng), dandelion (Pu gong ying), Tokyo violet (Zi hua di ding), Poria (Fu ling), wild chrysanthemum flower (Ye ju hua), Salvia (Dan shen), combined spicebush root (Wu yao) Modifications: Inflammation: safflower (Hong hua), Chinese wax gourd seed (Dong gua ren), Chinese honeylocust spine (Zao jiao ci) Chest oppression: *Bupleurum* (Chai hu), long stamen onion bulb (Xie bai), snake gourd fruit (Gua lou) Severe phlegm-heat: Moutan (Dan pi), weeping forsythia capsule (Lian qiao)	Not mentioned blindness and drop-out rate

[24]	RCT	96	Treatment arm: Yiqi Huayu Penyan Decoction + levofloxacin + Guizhi Fuling capsules Control arm: levofloxacin + Guizhi Fuling capsules	Treatment arm: TFR, 95.83% (46 of 48) Control arm: TFR, 83.33%^*∗*^ (40 of 48)	Yiqi Huayu Penyan Decoction: Radix codonopsis (Dang shen), Astragalus (Huang qi), *Angelica sinensis* (Dang gui), Salvia (Dan shen), *Ligusticum wallichii* (Chuan xiong), Cyperus (Xiang fu), *Liquidambar formosana* Hance (Lu lu tong), white peony root (Bai shao), leech (Shuizhi), licorice (Gancao) Modifications: Abnormal vaginal discharge: add heartleaf houttuynia (Yu xing cao), bearded scutellaria (Ban zhi lian), Amur cork-tree bark (Huang bai) Pruritus vulvae: add Coix seed (Yi yi ren), Hypoglaucous Collett Yam Rhizome (Bi xie) Diarrhea: add Psoralea (Bu gu zhi), common yam rhizome (Shan yao)	Not mentioned blindness and drop-out rate

[25]	RCT	108	Treatment arm: Dahuangmudan Decoction + cefoxitin + doxycycline Control arm: cefoxitin + doxycycline	Treatment arm: TFR, 94.55% (52 of 55) Control arm: TFR, 79.25%^*∗*^ (42 of 53)	Dahuangmudan Decoction: rhubarb root and rhizome (Da huang), Moutan (Mu dan pi), Semen Persicae (Tao ren), Chinese wax gourd seed (Dong gua zi), sodium sulfate (Mang xiao), Herba Patriniae (Bai jiang cao), Delavay honeysuckle (Jin yin hua), red peony (Chi shao), Coix seed (Yi yi ren), licorice (Gan cao)	Not mentioned blindness and drop-out rate

[26]	RCT	100	Treatment arm: self-made Qingre Huayu Decoction + levofloxacin Control arm: levofloxacin	Treatment arm: TFR, 94% (47 of 50) Control arm: TFR, 80% ^*∗*^ (40 of 50)	Self-made Qingre Huayu Decoction: licorice (Gan cao), Amur cork-tree bark (Huang bai), Semen Persicae (Tao ren), Chinese wax gourd seed (Dong gua zi), Herba Leonuri (Yi mu cao), Corydalis tuber (Yan hu suo), Indian bread (Fu ling), Danshen root (Dan shen), red peony root (Chi shao), Sargentodoxa cuneata (Hong teng), *Patrinia* (Bai jiang cao), Tokyo violet (Zi hua di ding), dandelion (Pu gong ying), Delavay honeysuckle (Jin ying hua)	Not mentioned blindness and drop-out rate

[27]	RCT	118	Treatment arm: Fuyanshu capsule + levofloxacin hydrochloride + metronidazole Control arm: Fuyanshu capsule simulator + levofloxacin hydrochloride + metronidazole	Treatment arm: RR, 18.64% (11 of 59) Control arm: RR, 86.44%^*∗*^ (51 of 59)	Fuyanshu capsule: honeysuckle stem (Ren dong teng), Sargent gloryvine stem (Da xue teng), licorice (Gan cao), woad root (Da qing ye), dandelion (Pu gong ying), red peony root (Chi shao), rhubarb root and rhizome (Da huang), Danshen root (Dan shen), giant knotweed rhizome (Hu zhang), Toosendan fruit (Chuan lian zi), Corydalis tuber (Yan hu suo)	Not mentioned blindness and drop-out rate

[28]	RCT	110	Treatment arm: Danbai granules + azithromycin Control arm: azithromycin	Treatment arm: TFR, 98.2% (54 of 55) Control arm: TFR, 90.9%^*∗*^ (50 of 55)	Danbai granules: white peony root (Bai shao), Moutan (Mu dan pi), Tokyo violet (Zi hua di ding), common burr reed tuber (San leng), Ailanthus bark or root bark (Chuan pi), *Patrinia* root (Mu tou hui), *Patrinia* (Bai jiang cao), *Sparganium* (San leng), Curcumae rhizome (E zhu), Nightshade (Bai ying), Sichuan lovage root (Chuan xun), Sargent gloryvine stem (Da xue teng), glabrous greenbrier rhizome (Tu fu ling), Chinese Angelica (Dang gui), *Oldenlandia* (Bai hua she she cao)	Not mentioned blindness and drop-out rate

[29]	RCT	96	Treatment arm: Fuyanning Decoction + levofloxacin Control arm: levofloxacin	Treatment arm: TFR, 97.92% (47 of 48) Control arm: TFR, 87.50%^*∗*^ (42 of 48)	Fuyanning Decoction: Danshen root (Dan shen), Astragalus root (Huang qi), Myrrh (Mo yao), Sargentodoxa cuneata (Hong teng), Dodder seed (Tu si zi), Morinda root (Ba ji tian), heartleaf houttuynia (Yu xing cao), flying squirrel faeces (Wu ling zhi), Herba Patriniae (Bai jiang cao), white Atractylodes rhizome (Bai zhu), degelatinated deer antler powder (Lu jiao shaung), Coix seed (Yi yi ren), Indian bread (Fu ling), Lychee seed (Li zhi he), Cassia twig (Gui zhi), Licorice (Gan cao)	Not mentioned blindness and drop-out rate

[30]	RCT	120	Treatment arm: Guizhi Fuling pills + ornidazole Control arm: ornidazole	Treatment arm: TFR, 91.67% (55 of 60) Control arm: TFR, 78.33%^*∗*^ (47 of 60)	Guizhi Fuling pills: *Cassia* twig (Gui zhi), Indian bread (Fu ling), Moutan (Mu dan pi), red peony root (Chi shao), Semen Persicae (Tao ren)	Not mentioned blindness and drop-out rate

[31]	RCT	155	Treatment arm: the first 14 days: placebo (levofloxacin + metronidazole) + Jinying capsules; remaining 14 days: Jinying capsules Control arm: the first 14 days: Jinying capsules placebo + levofloxacin + metronidazole; remaining 14 days: Jinying capsule placebo	Treatment arm: TFR, 76.32% (60 of 78) Control arm: TFR, 59.46%^*∗*^(46 of 77)	Jinying capsules: Delavay honeysuckle (Jin yin hua), Amur cork-tree bark (Huang Bai), Tokyo violet (Zi hua di ding), wild chrysanthemum flower (Ye ju hua), dandelion (Pu gong ying), Atractylodes rhizome (Cang zhu), Chinese honeylocust spine (Zao jiao ci), Salvia (Dan shen), red peony (Chi shao), Corydalis tuber (Yan hu suo)	Not mentioned blindness and drop-out rate

[32]	RCT	38	Treatment arm: Penning granule Control arm: levofloxacin + metronidazole	Treatment arm: TFR, 91.3% (21 of 23) Control arm: TFR, 60%^*∗*^ (9 of 15)	Penning granule: Sargent gloryvine stem (Da xue teng), Patrinia (Bai jiang cao), *Oldenlandia* (Bai hua she she cao), red peony (Chi shao), *Angelica sinensis* (Dang gui), Frankincense (Ru Xiang), Myrrh (Mo yao), common burr reed tuber (San leng), Curcumae rhizome (E zhu), Chinese honeylocust spine (Zao jiao ci), Salvia (Dan shen), *Bupleurum* (Chai hu), Manchurian wild ginger (Xi xin), Astragalus (Huang qi), Pangolin scales (Chuan shan jia)	Not mentioned blindness, drop-out rate, and small sample size

[33]	RCT	90	Tongluo Qingre Decoction arm GeXia Zhuyu decoction arm	Tongluo Qingre Decoction arm: TFR, 91.11% (41 of 45) GeXia Zhuyu Decoction arm: TFR, 82.22% ^*∗*^ (37 of 45)	Tongluo Qingre Decoction: giant knotweed rhizome (Hu zhang), glabrous greenbrier rhizome (Tu fu ling), Amur cork-tree bark (Huang bai), ground beetle (Tu bie chong), Red peony (Chi shao), Moutan (Mu dan pi), Semen Persicae (Tao ren), Sichuan lovage root (Chuan xiong), Toosendan fruit (Chuan lian zi), leech (Shui zhi), Chinese Angelica (Dang gui), safflower (Hong hua), turmeric root tuber (Yu jin) GeXia Zhuyu Decoction: flying squirrel faeces (Wu ling zhi), Chinese Angelica (Dang gui), Sichuan lovage root (Chuan xiong), Semen Persicae (Tao ren), Moutan (Mu dan pi), red peony (Chi shao), combined spicebush root (Wu yao), Corydalis tuber (Yan hu suo), licorice (Gan cao), Cyperus (San leng cao), nutgrass galingale rhizome (Xiang fu), safflower (Hong hua), bitter orange (Zhi qiao)	Not mentioned blindness and drop-out rate

[34]	RCT	180	Treatment arm: pelvic inflammatory decoction Decoction arm: physical therapy Physiotherapy arm: inflammatory decoction + physical therapy	Treatment arm: TFR, 96.3% (59 of 60) ^*∗*^ decoction arm: TFR, 86.7% (52 of 60) Physiotherapy arm: TFR, 76.7% (46 of 60)	Pelvic inflammatory decoction: Atractylodes rhizome (Cang zhu), Astragalus (Huang qi), licorice (Gan cao), Radix codonopsis (Dang shen), *Liquidambar formosana* Hance (Lu lu tong), weeping forsythia capsule (Lian qiao), Platycodon root (Jie geng), combined spicebush root (Wu yao), common self-heal fruit-spike (Xia ku cao), Delavay honeysuckle (Jin yin hua)	Not mentioned blindness and drop-out rate

[35]	RCT	80	Treatment arm: Shaofu Zhuyu Decoction + tinidazole + levofloxacin Control arm: tinidazole + levofloxacin	Treatment arm: TFR, 95% (38 of 40) Control arm: TFR, 80%^*∗*^(32 of 40)	Shaofu Zhuyu Decoction: Fennel (Xiao hui xiang), dried ginger rhizome (Gan jiang), Corydalis tuber (Yuan hu), Trogopterus dung (Wu ling zhi), *Ligusticum wallichii* (Chuan xiong), Cattail Pollen (Pu huang), *Angelica sinensis* (Dang gui), red peony (Chi shao), cinnamon bark (Rou gui) Modifications: Pain: Toosendan fruit (Chuan lian zi) Chest oppression: Cyperus (Xiang fu), turmeric root tuber (Yu jin) Waist pain: *Eucommia* (Du zhong), Psoralea (Bu gu zhi) Lack of strength: Radix codonopsis (Dang shen), *Astragalus* (Huang qi)	Not mentioned blindness, drop-out rate, and small sample size

*Note.* RCT: randomized clinical trial; TFR: total effective rate; RR: recurrence rate. ^*∗*^*p* < 0.05 versus treatment arm.

**Table 2:** The RCTs of effective CHMRE for PID.

Study ID	Design	Sample size	Interventions	Outcomes	Composition	Limitation
[44]	RCT	184	Control arm: abdominal ultrasound drug delivery therapy Treatment arm: CHM staining therapy combined with CHMRM	Control arm: TFR, 91.95%^*∗*^ (88 of 92) Treatment arm: TFR, 97.82% (90 of 92)	CHMRE prescription: common burr reed tuber (San leng), Curcumae rhizome (E zhu), Corydalis tuber (Yan hu suo), Toosendan fruit (Chuan lian zi), Cassia twig (Gui zhi), Sargentodoxa cuneata (Hong teng), Herba Patriniae (Bai jiang cao), Delavay honeysuckle (Jin yin hua), weeping forsythia capsule (Lian qiao), Poria (Fu ling)	Not mentioned drop-out rate

[45]	RCT	110	Control arm: cefotaxime sodium + metronidazole + fluconazole (mycotic infection)/doxycycline (mycoplasma infection) Treatment arm: combined with enema Chinese prescription on the basis of the control arm	Control arm: TFR, 61.82%^*∗*^ (34 of 55) Treatment arm: TFR, 90.91% (34 of 55)	Enema Chinese prescription: Herba Patriniae (Bai jiang cao), Salvia (Dan shen), Tokyo violet (Zi hua di ding), dandelion (Pu gong ying), Toosendan fruit (Chuan lian zi), heartleaf houttuynia (Yu xing cao), red peony (Chi shao) Modifications: Abdominal distention: Semen Vaccariae (Wang bu liu xing), *Liquidambar formosana* Hance (Lu lu tong) Cold-damp constraining: combined spicebush root (Wu yao), Poria (Fu ling), Cassia twig (Gui zhi)	Not mentioned blindness and drop-out rate

[47]	RCT	90	Control arm: levofloxacin + ornidazole Treatment arm: self-made Gynecological Anti-Inflammatory No. 1 Decoction on the basis of the control arm	Control arm: TFR, 75.56%^*∗*^ (34 of 45) Treatment arm: TFR, 93.33% (42 of 45)	Self-made Gynecological Anti-Inflammatory No.1 Decoction: Delavay honeysuckle (Jin yin hua), dandelion (Pu gong ying), weeping forsythia capsule (Lian qiao), Oldenlandia (Bai hua she she cao), Herba Patriniae (Bai jiang cao), heartleaf houttuynia (Yu xing cao), Sargent gloryvine stem (Da xue teng), red peony (Chi shao), Moutan (Mu dan pi), cinnamon bark (Rou gui), green tangerine peer (Qing pi), Morinda root (Ba ji tian), white Atractylodes rhizome (Bai zhu), citrus (Chen pi), Coix seed (Yi yi ren), Poria (Fu ling)	Not mentioned drop-out rate and small sample size

[48]	RCT	78	Control arm: levofloxacin + ornidazole Treatment arm: self-made pelvic inflammation decoction (oral and retention enema)	Control arm: TFR, 79.49%^*∗*^ (31 of 39) Treatment arm: TFR, 92.31% (36 of 39)	Self-made pelvic inflammation decoction: Sargentodoxa cuneata (Hong teng), white Atractylodes rhizome (Bai zhu), Euryale seed (Qian shi), common yam rhizome (Huai shan), Dragon bones (Long gu), Concha Ostreae (Mu li), Radix Pulsatillae (Bai tou weng), Haematitum (Dai zhe shi), Dodder seed (Tu si zi), heartleaf houttuynia (Yu xing cao), dandelion (Pu gong ying), Angelica root (Bai zhi), cuttlebone (Hai piao xiao), Radix et Rhizoma Rubiae (Qian cao), Semen Plantaginis (Che qian zi), Semen Ginkgo (Bai guo), Amur cork-tree bark (Huang bai)	Not mentioned drop-out rate and small sample size

[49]	Case-control study	70	Control arm: Oral Xiaoyan Decoction Treatment arm: Xiaoyan Decoction (oral and retention enema)	Control arm: TFR, 80.00%^*∗*^ (28 of 35) Treatment arm: TFR, 97.14% (34 of 35)	Xiaoyan Decoction: Cyperus (Xiang fu), Radix Bupleuri (Cai hu), *Ligusticum wallichii* (Chuan xiong), red peony (Chi shao), *Angelica sinensis* (Dang gui), Corydalis tuber (Yan hu suo), Rhizoma Cibotii (Gou ji), Psoralea (Bu gu zhi), Sargentodoxa cuneata (Hong teng), Herba Patriniae (Bai jiang cao), Dandelion (Pu gong ying), white Atractylodes rhizome (Bai zhu), Semen Euryales (Qian shi), fresh ginger (Sheng jiang)	Not mentioned blindness, drop-out rate, and small sample size

[51]	RCT	92	Control arm: tubal hydrotubation Treatment arm: pelvic inflammation recipe (oral and retention enema)	Control arm: TFR, 80.43^*∗*^ (37 of 46) Treatment arm: TFR, 95.65% (44 of 46)	Pelvic inflammation recipe: *Liquidambar formosana* Hance (Lu lu tong), red peony (Chi shao), Sargentodoxa cuneata (Hong teng), Herba Patriniae (Bai jiang cao), Caulis Lonicerae Japonicae (Ren dong teng), Tokyo violet (Zi hua di ding), lychee seed (Li zhi he)	Not mentioned drop-out rate and small sample size

[53]	RCT	50	Control arm: laparoscopic surgery Treatment arm: combined with Pen Yan Qing on the basis of the control arm	Control arm: TFR, 52% ^*∗*^ (13 of 25) Treatment arm: TFR, 84% (21 of 25)	Pen Yan Qing: Danshen root (Dan shen), common burr reed tuber (San leng), Curcumae rhizome (E zhu), Sargentodoxa cuneata (Hong teng) (because the drug is applied for a new drug, the full prescription is not disclosed)	Not mentioned drop-out rate and small sample size

[54]	RCT	86	Control arm: antibiotic Treatment arm: combined with CHMRE on the basis of the control arm	Control arm: PR, 41.8% ^*∗*^ treatment arm: PR, 62.7%	CHMRE: Delavay honeysuckle (Jin yin hua), Sargentodoxa cuneata (Hong teng), weeping forsythia capsule (Lian qiao), Chinese honeylocust spine (Zao jiao ci), Patrinia (Bai jiang cao), Amur cork-tree bark (Huang bai), Moutan (Mu dan pi), Frankincense (Ru xiang), Myrrh (Mo yao), Curcumae rhizome (E zhu), Corydalis tuber (Yan hu suo)	Not mentioned drop-out rate and small sample size

[55]	RCT	74	Control arm: ceftizoxime sodium/levofloxacin lactate + ornidazole Treatment arm: combined with CHMRE on the basis of the control arm	Control arm: TFR, 75.68%^*∗*^ (28 of 37) Treatment arm: TFR, 94.59% (35 of 37)	CHMRE: Radix et Rhizoma Cynanchi Paniculata (Xu chang qing), Chinese honeylocust spine (Zao jiao ci), Sargentodoxa cuneata (Hong teng), Dandelion (Pu gong ying), Delavay honeysuckle (Jin yin hua), Herba Patriniae (Bai jiang cao), Danshen root (Dan shen), Radix Scutellariae (Huang qin)	Not mentioned drop-out rate and small sample size

*Note.* RCT: randomized clinical trial; TFR: total effective rate; RR: recurrence rate, PR: pregnancy rate. ^*∗*^*P* < 0.05 versus treatment arm.

**Table 3:** The location, regional anatomy, and innervation of common acupoints for treating PID.

Acupoint	Location	Muscle	Innervation
CV4 (Guanyuan)	3 cun below the center of the umbilicus on the lower abdomen and on the anterior midline	Fibrous tissue, linea alba	L1
SP6 (Sanyinjiao)	3 cun proximal to the medial malleolus	Mm. flexor digitorum longus, tibialis posterior	L4-5, S1-2
EX-CA1 (Zigong)	4 cun below the umbilicus and 3 cun lateral to the anterior midline	Obliquus internus abdominis, musculus transversus abdominis	T10-L2
CV3 (Zhongji)	4 cun caudal to the umbilicus	Fibrous tissue, linea alba	L1
ST36 (Zusanli)	3 cun below ST35, one finger breadth from the anterior crest of the tibia, (front ridge of tibia), between fibula and tibia	Anterior tibial muscle, extensor digitorum longus	L4-5, S1-2
CV6 (Qihai)	1.5 cun inferior to the center of the umbilicus, on the anterior midline	Fibrous tissue, linea alba	Th11
ST29 (Guilai)	4 cun inferior to the center of the umbilicus, 2 cun lateral to the anterior midline	M. rectus abdominis	Th6-12
BL32 (Ciliao)	At the 2nd posterior sacral foramen on the sacrum and the posterior ramus of the S2 nerve	Erector spinae	L2-S4
BL23 (Shenshu)	Under the 2nd spinous process of lumbar vertebra, next to 1.5 cun	Erector spinae	L1
SP9 (Yinlingquan)	Below medial tibia condyle	M. gastrocnemius	S1-2

## Data Availability

No data were used to support this study.
